# EA-UNET: An Enhanced and Efficient Model for Left-Turn Lane

**DOI:** 10.3390/s26092642

**Published:** 2026-04-24

**Authors:** Haowei Wang, Haixin Liu, Fei Wang, Xingbin Chen, Baogang Li, Jiang Liu

**Affiliations:** 1 School of Mechanical and Automotive Engineering, Qingdao University of Technology, Qingdao 266520, China; wangyushun@qut.edu.cn (H.W.); wangfeiernv@163.com (F.W.); cxb19862003@163.com (X.C.); baog_li@alu.cqu.edu.cn (B.L.); 2 Guangdong Productivity Promotion Center, Guangzhou 510075, China

**Keywords:** autonomous vehicle, left-turn lanes, lightweight network, EA-UNet, Convolutional Block Attention Module

## Abstract

Left-turn lanes are critical elements of urban intersections. Accurate and efficient lane detection is essential for the safe navigation of autonomous vehicles. To address the limitations of existing semantic segmentation algorithms—specifically, inadequate detection accuracy, high computational cost, and vulnerability to environmental disturbances—we propose a lightweight deep convolutional neural network named EA-UNet. First, we replace the standard U-Net encoder with EfficientNet-B0 to enhance feature extraction efficiency. Second, we introduce a novel contextual coordination module, termed MP-ASPP, which integrates a Convolutional Block Attention Module (CBAM) to further refine attention mechanisms. Finally, a comprehensive real-world dataset was constructed by collecting videos and images of left-turn waiting areas during real-vehicle testing. Experimental results demonstrate that EA-UNet significantly outperforms the baseline U-Net and other state-of-the-art models, achieving accurate and efficient segmentation of left-turn lanes even in complex scenes.

## 1. Introduction

Left-turn lanes represent critical and complex scenarios at urban intersections. Navigating these environments poses significant challenges for autonomous vehicles due to intricate traffic conditions, diverse road markings, and strict driving regulations. Consequently, the accurate recognition and assessment of the road environment constitute the foundational step for safe autonomous navigation in left-turn scenarios. Traditional road recognition methods rely on computer vision techniques and image processing algorithms, and it is difficult for them to meet the robustness requirements of automatic driving road recognition. Kong et al. [1] used Gabor filters to extract texture orientation and employed a local soft voting strategy to estimate vanishing points (Kong et al., 2010). Fang et al. [2] utilized color features for global oversampling and completed the road segmentation task by merging oversampled regions (Fang et al., 2010). Wu and Duan [3] applied the RGB entropy method to process road images and extracted road areas using an improved region growing approach (Wu and Duan, 2019). Chen et al. [4] proposed a method that utilizes a Kalman filter for tracking to dynamically determine the lane line region. Although these methods can successfully extract the road areas in specific scenarios, they are easily interfered with by external environmental factors and cannot meet the requirements of high robustness for automatic driving road recognition.

With the development of deep learning, road recognition methods tend to adopt convolutional neural networks, including FCN (Long et al., 2015) [5], SegNet (Badrinarayanan et al., 2017) [6], Unet (Ronneberger et al., 2015) [7], PSPNet (Zhao et al., 2017) [8], DeepLabV3+ (Chen et al., 2018) [9], and others. Compared with traditional segmentation algorithms, these algorithms have higher robustness in segmentation performance and are relatively fast. The UNet proposed by Ronneberger et al. employs a fully symmetric encoder–decoder structure, concatenating and fusing features from deep and shallow networks to enhance segmentation accuracy. However, it tends to face sampling edge information loss due to its multiple down and up operations. An improved UNet++ model redesigns skip connections by introducing dense blocks and convolutional layers between the encoder and decoder to reduce the sampling loss (Zhou et al., 2018) [10]. However, its convolutional network upsampling would result in blurry smoothness, making it insensitive to details in image regions. 3D-Unet [11] model replaces all 2D convolutions in UNet with 3D convolution blocks, incorporating Batch Normalization to prevent gradient explosions (Çiçek et al., 2016). But it may limit the segmenting for small objects or low-contrast regions. In order to focus on interest regions, Attention-Unet introduces attention mechanisms in the decoder part (Oktay et al., 2018) [12]. Nevertheless, its robustness in complex situations may be insufficient. In pursuit of a more lightweight model, Rail-Net proposed by Li and Peng exhibits good detection speed but relatively lower accuracy (Li and Peng, 2022) [13]. The DFA-Unet network, proposed by Zhang et al. [14], utilizes the same encoder–decoder structure as UNet but enhances accuracy by embedding DFA for the encoder (Zhang et al., 2023). TransUNet incorporates Transformer structures into the UNet network through self-attention mechanisms for feature extraction and prediction, capturing global dependencies for improved feature representation and accuracy (Chen et al., 2021) [15]. CENet aggregates context clues by densely upsampling features from the encoder layer to the decoder layer, aiming to capture multi-scale context information (Zhou et al., 2022) [16]. R2U++ enhances critical features by replacing ordinary convolution blocks in U-Net with recurrent residual convolutions (Mubashar et al., 2022) [17]. AC-Unet extracts features by adding cross convolutions and attention mechanisms, simultaneously introducing a Feature Refinement Module (FRM) to learn global context information (Fan et al., 2023) [18]. The above methods mainly concern pixel segmentation accuracy metrics. The accuracy alone is not enough for autonomous driving in turning-lane scenes; model size and algorithm complexity have to be considered.

## 2. Related Work

To address these limitations, we propose EA-UNet, an enhanced and lightweight model specifically optimized for left-turn lane detection. Our study aims to contribute to the field in the following ways: In summary, this paper aims to advance the field through the following key contributions:

Lightweight Network Design: We develop EA-UNet, an optimized semantic segmentation model. By employing an EfficientNet-based structure as the encoder and streamlining high-level skip connections, the model drastically minimizes computational complexity and parameter overhead.

Enhanced Feature Extraction and Edge Refinement: To address semantic ambiguity in complex environments, we introduce a context coordination module (MP-ASPP) coupled with a Convolutional Block Attention Module (CBAM). This integration significantly boosts multi-scale feature representation and sharply delineates critical lane boundaries.

Rigorous Real-world Validation: We introduce a dedicated dataset comprising real-world left-turn waiting lane scenarios collected by vehicle-mounted cameras. Comprehensive empirical evaluations validate that the proposed EA-UNet consistently surpasses mainstream baselines—including U-Net, DeepLabV3+, and PSPNet—in terms of both accuracy and efficiency.

It is worth noting that left-turn waiting lane segmentation targets a fine-grained intersection semantics, where accurate labeling typically requires delineating the waiting region boundary and its spatial relationship with directional markings and intersection-specific lane configurations. Mainstream public driving datasets often provide lane markings, drivable-area masks, or generic semantic categories, but do not explicitly define “left-turn waiting regions” as a dedicated pixel-level class, which makes direct benchmarking under the same task definition non-trivial without additional scenario filtering and relabeling (many benchmarks are oriented toward lane detection or lane-line annotation).

At present, public benchmarks tailored for the semantic segmentation of left-turn waiting areas remain unavailable in the field. This specific task necessitates fine-grained, pixel-level annotations of complex intersection topologies and directional markings—features that are notably absent from mainstream autonomous driving datasets. To bridge this critical gap, we curated a novel, task-specific dataset captured from diverse real-world urban intersections. By providing rigorous annotations tailored to these specialized road structures, our dataset facilitates a robust and highly targeted empirical evaluation of the proposed methodology under realistic driving conditions.

To address this gap, we construct a dedicated dataset from real-world traffic videos and employ geometric/photometric as well as adverse-weather/occlusion-oriented augmentations and negative samples to improve robustness and reproducibility; details are provided in the Data Collection and Processing subsection.

## 3. Materials and Methods

### 3.1. Overall Model Design

**Figure 1:**
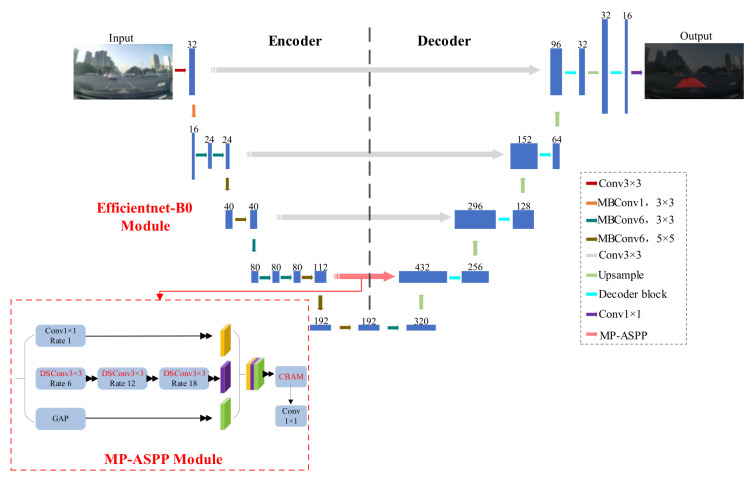
EA-UNet network and MP-ASPP module.

This paper proposes an enhanced segmentation network, EA-UNet, which introduces two significant improvements to the standard U-Net architecture. First, the conventional U-Net encoder is replaced with EfficientNet-B0 to serve as a lightweight backbone. This optimization strategy significantly enhances the operational efficiency of the model. Second, a novel MP-ASPP module is introduced within the high-level skip connections. Because direct concatenation between the encoding and decoding paths in the original U-Net can lead to the loss of fine-grained details, the MP-ASPP module replaces standard dilated convolutions with dilated depthwise separable convolutions and integrates a Convolutional Block Attention Module (CBAM) (Woo et al., 2018) [19]. This strategic integration effectively captures more detailed contextual features and preserves the edge information of the target objects, thereby improving overall segmentation accuracy. The complete architecture of the proposed EA-UNet is illustrated in Figure 1.

### 3.2. EfficientNet-B0 Network Optimization

Conventional convolutional neural network (CNN) architectures typically enhance performance by scaling one of three dimensions: input resolution, network depth, or channel width. This empirical tuning process necessitates extensive manual adjustments, thereby complicating network design and significantly increasing computational overhead. To overcome these limitations, Tan and Le [20] proposed EfficientNet, a lightweight architecture that achieves adaptive optimization across width, depth, and resolution via a unified compound scaling factor. Among the EfficientNet series (B0 to B7), EfficientNet-B0 features the smallest parameter count and the fastest inference speed, making it the optimal choice as the encoder for our proposed model.

The standard EfficientNet-B0 comprises two convolutional layers, 16 Mobile Inverted Bottleneck Convolution (MBConv) blocks, a global average pooling (GAP) layer, and a fully connected (FC) layer. Crucially, each MBConv block incorporates a squeeze-and-excitation (SE) network. The SE module performs two-dimensional global average pooling to compress high-dimensional feature maps into low-dimensional feature vectors, effectively extracting channel-wise global features through squeeze-and-excitation operations. Although SE modules utilize a multilayer perceptron for nonlinear feature transformation, they focus exclusively on encoding inter-channel dependencies. This neglect of crucial spatial and positional information limits the model’s recognition performance, particularly in complex and highly structured scenarios like left-turn lanes.

**Figure 2:**
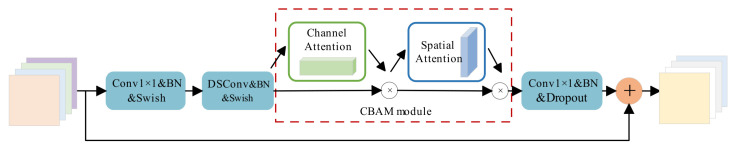
Improved MBConv convolution block structure.

To address this deficiency, we propose targeted optimizations to both the overall EfficientNet architecture and its constituent MBConv blocks. By strengthening the learning of positional information without sacrificing the lightweight nature of the network, we enhance the spatial feature extraction capabilities. The specific optimization process is detailed as follows:

Integration of the CBAM Attention Mechanism: We replace the standard SE module within the MBConv block with a Convolutional Block Attention Module (CBAM). Unlike the SE module, which focuses solely on channel dependencies, CBAM infers attention maps sequentially along both channel and spatial dimensions. This integration ensures that the network accurately focuses on the left-turn lane regions while preserving precise spatial and positional information. The architecture of the improved MBConv block is illustrated in Figure 2.

Network Lightweighting: Deep convolutional networks often suffer from high parameter counts during successive downsampling. To enhance feature extraction efficiency while reducing computational complexity, we strategically decrease the number of MBConv blocks. Specifically, four MBConv layers were removed from the original architecture, retaining a streamlined sequence of 12 layers. This modification further lightens the overall network structure without compromising segmentation accuracy. The revised architecture of the enhanced EfficientNet encoder is detailed in Table 1.

The EfficientNet architecture is primarily constructed by stacking multiple MBConv (Mobile Inverted Bottleneck Convolution) modules. The MBConv module was originally introduced in MobileNetV2 and subsequently refined in EfficientNet. Specifically, the original ReLU6 activation function is replaced with the Swish activation function to improve nonlinear representation capability. In addition, a squeeze-and-excitation (SE) module is incorporated into each MBConv block to enhance channel-wise feature recalibration. These modifications enable more effective feature extraction and significantly improve the representational capacity of the network.

### 3.3. MP-ASPP Module

**Figure 3:**
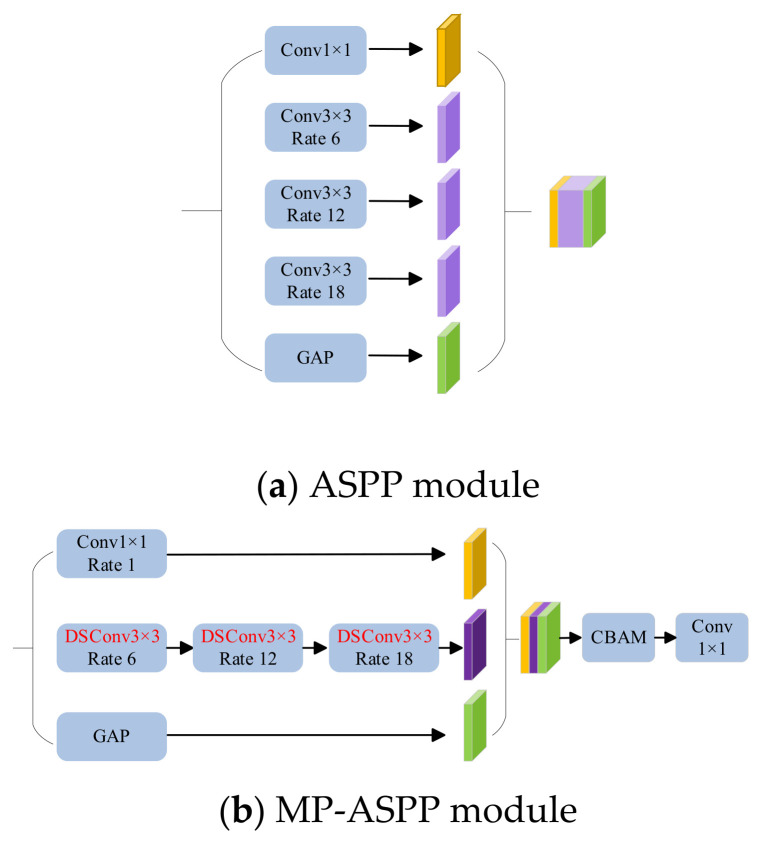
ASPP module and MP-ASPP module.

The Atrous Spatial Pyramid Pooling (ASPP) module, originally proposed in the DeepLab series, employs five parallel branches to extract multi-scale context. The first branch utilizes a standard 1 × 1 convolution, while the subsequent three branches employ 3 × 3 atrous (dilated) convolutions with varying dilation rates to extract multi-scale information. The final branch introduces global average pooling (GAP) to capture global image context. By concatenating the outputs of these five branches along the channel dimension, ASPP effectively captures multi-scale target information, mitigating local information loss and correlating distant features. The standard ASPP structure is illustrated in Figure 3.

However, the parallel computation across multiple branches significantly increases computational complexity and lacks inter-branch connectivity. Inspired by the ASPP architecture, we propose a novel Multi-Branch Series-Parallel Atrous Spatial Pyramid Pooling (MP-ASPP) module. This module integrates depthwise separable convolutions and the CBAM attention mechanism, as depicted in Figure 3.

By embedding the MP-ASPP module within the high-level skip connections of EA-UNet, the network effectively captures rich, multi-scale features while minimizing computational overhead. This integration significantly enhances the extraction of crucial information from the feature maps. The specific architectural improvements are outlined as follows:

Integration of Depthwise Separable Convolutions: Standard 3 × 3 dilated convolutions tend to learn redundant information, leading to high parameter counts and prolonged training times. To address this, we replace the standard convolutions in the three parallel branches with depthwise separable convolutions, which decompose a standard convolution into depthwise and pointwise convolutions. This method significantly reduces floating-point operations (FLOPs) and parameters with minimal impact on accuracy. Furthermore, these three branches are reconfigured into a serial connection. This series-parallel arrangement effectively reduces the dimensionality of each branch, further decreasing the computational load and complexity of the module.

Incorporation of the CBAM Attention Mechanism: To compensate for the potential slight decrease in segmentation accuracy caused by depthwise separable convolutions, we incorporate the CBAM attention mechanism at the final layer of the MP-ASPP module. CBAM dynamically allocates attention resources across both spatial and channel dimensions within the high-level skip connections of EA-UNet. This effectively suppresses irrelevant background regions, captures finer details, preserves critical edge information of the target objects, and ultimately enhances overall segmentation accuracy.

**Figure 4:**
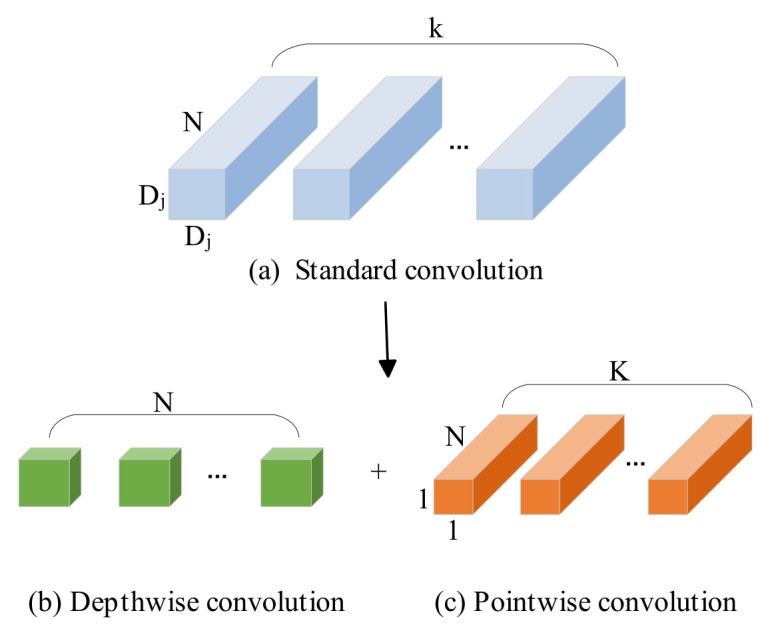
Standard convolution and depthwise separable convolution.

Depthwise separable convolution is a convolutional method that utilizes depthwise separable convolution to perform separate computations on channels and spatial dimensions. It consists of two parts: depthwise convolution and pointwise convolution, as illustrated in Figure 4.

Assuming the input feature map has dimensions $D_{i}\times D_{i}$, with N channels, a convolutional kernel size of $D_{j}\times D_{j}$, and an output feature map with K channels, the corresponding computational cost $Q_{1}$ and parameter count $P_{1}$ for standard convolution are as follows:

(1) $$Q_{1}=D_{i}\times D_{i}\times N\times K\times D_{j}\times D_{j}$$

(2) $$P_{1}=N\times K\times D_{j}\times D_{j}$$

The computational cost $Q_{2}$ and parameter count $P_{2}$ for depthwise separable convolution are as follows:

(3) $$Q_{2}=D_{i}\times D_{i}\times N\times D_{j}\times D_{j}+N\times K\times D_{i}\times D_{i}$$

(4) $$P_{2}=N\times D_{j}\times D_{j}+N\times K$$

Hence, the ratio between the computational cost and parameter count of the two can be obtained:

(5) $$\frac{Q_{2}}{Q_{1}}=\frac{D_{i}\times D_{i}\times N\times D_{j}\times D_{j}+N\times K\times D_{i}\times D_{i}}{D_{i}\times D_{i}\times N\times K\times D_{j}\times D_{j}}=\frac{1}{K}+\frac{1}{D_{j^{2}}}$$

(6) $$\frac{P_{2}}{P_{1}}=\frac{N\times D_{j}\times D_{j}+N\times K}{N\times K\times D_{j}\times D_{j}}=\frac{1}{K}+\frac{1}{D_{j^{2}}}$$

For example, in the case of a common structure with 112 channels and a 3 × 3 convolutional kernel, the ratio of Q to P for both is approximately 0.12. Therefore, it can be inferred that depthwise separable convolution achieves a significant reduction in computational cost and parameter count. By replacing dilated convolution with depthwise separable convolution and introducing the CBAM attention mechanism, the MP-ASSP module gains a more extensive receptive field. It can provide the capability to densely utilize multi-scale information, balancing the practical demands of parameter size and feature representation.

## 4. Experiments and Analysis

### 4.1. Data Collection and Processing

**Figure 5:**
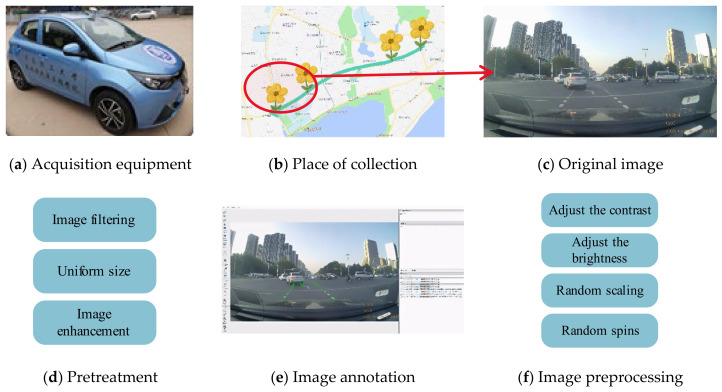
Image acquisition and processing process.

The overall image acquisition and processing workflow is illustrated in Figure 5. The primary dataset was collected along the Changjiang Road section in Huang dao District, Qingdao, China. Video footage was captured at various intersections under diverse lighting and weather conditions—including sunny, cloudy, and nighttime environments—as well as varying degrees of occlusion from preceding vehicles. From these videos, frames containing left-turn waiting areas were systematically extracted to construct the initial dataset.

**Figure 6:**
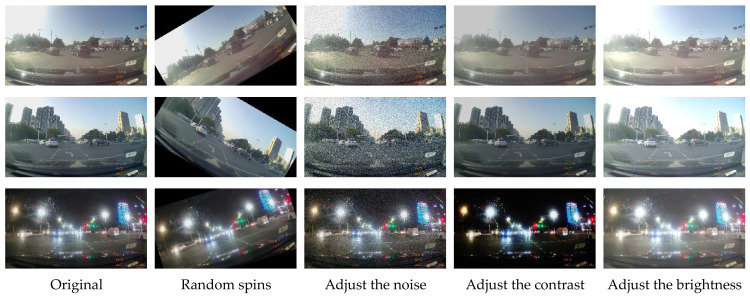
Example of data enhancement.

**Figure 7:**
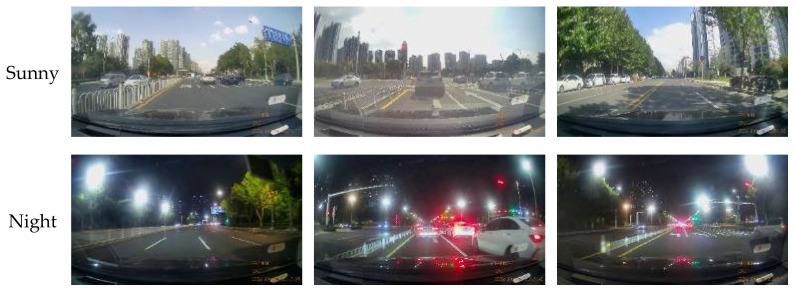
Negative sample example.

To enhance the model’s generalization capabilities and mitigate overfitting, the extracted images were subjected to several data augmentation techniques, including adjustments to contrast and brightness, as well as random rotation, cropping, and scaling. Representative examples of these augmentation techniques are shown in Figure 6. Furthermore, 200 negative samples (images without left-turn lanes) were included without augmentation, yielding a final dataset comprising 2400 images. Examples of these negative samples are provided in Figure 7. During this phase, all input images are uniformly processed to 256 × 256 pixels in size [21].

The complete dataset was randomly divided into a training set and a validation set using a 9:1 ratio. The training set was utilized to optimize the model parameters, while the validation set was employed to monitor generalization performance during the training process. To ensure dimensional consistency, all images were uniformly resized to 256 × 256 pixels and normalized prior to network input. Manual pixel-level annotation was performed using the LabelMe software to generate the ground-truth masks for semantic segmentation. The resulting annotations were exported in JSON format, capturing the precise coordinates of the polygon vertices and their corresponding category labels.

It is worth noting that there is currently no publicly available dataset specifically designed for left-turn waiting lane segmentation. This task involves highly specialized road structures and fine-grained semantic annotations (e.g., left-turn waiting regions and directional markings), which are not explicitly defined in existing public datasets. To address this limitation, a dedicated dataset was constructed based on real-world traffic scenes, focusing on complex intersection environments. During vehicle operation, various environmental factors should be considered, because elements such as nighttime conditions and varying lighting can significantly impede image-based lane detection [22]. The dataset provides pixel-level annotations tailored to the target task, enabling a more realistic and task-specific evaluation of the proposed method.

### 4.2. Experimental Setup and Evaluation Metrics

The proposed EA-UNet and all comparison models were implemented using the PyTorch 1.10.0 deep learning framework. To ensure a fair and rigorous evaluation, all training and testing procedures were conducted under identical hardware and software configurations, as detailed in Table 2 and Table 3.

The Adam optimizer was employed to update the network parameters, configured with a momentum of 0.9 and a weight decay of 0. To accelerate convergence and enhance overall model optimization, the network was trained for a total of 100 epochs using a two-stage training strategy. During the initial 50 epochs (the frozen stage), the backbone weights were frozen, and the batch size was set to 8. For the subsequent 50 epochs (the unfrozen, fine-tuning stage), the entire network was updated. To accommodate hardware memory constraints and prevent out-of-memory errors during this full-network training phase, the batch size was reduced to 4.

The foundational metrics for evaluating the model’s pixel-level classification performance include True Positive (TP), True Negative (TN), False Positive (FP), and False Negative (FN). Based on these components, this study utilizes the mean Intersection over Union (mIoU), Recall, Precision, and F1-score to comprehensively assess segmentation accuracy.

The mIoU represents the mean ratio of the intersection to the union of the predicted segmentation masks and the ground-truth labels across all classes. A higher mIoU value denotes superior spatial overlap between the predicted and actual regions, reflecting exceptional model performance. Recall measures the proportion of actual positive instances that are correctly identified by the model, while Precision evaluates the correctness of the positive predictions. The F1-score serves as the harmonic mean of Precision and Recall, providing a robust, single-metric evaluation for binary and multi-class segmentation tasks, especially when dealing with imbalanced datasets. The specific formulas for these calculations are as follows:

(7) $$\mathrm{MIOU}=\frac{\mathrm{TP}}{\mathrm{TP}+\mathrm{FN}+\mathrm{FP}}$$

(8) $$\mathrm{Precision}=\frac{\mathrm{TP}}{\mathrm{TP}+\mathrm{FP}}$$

(9) $$\mathrm{Recall}=\frac{\mathrm{TP}}{\mathrm{TP}+\mathrm{FN}}$$

(10) $$F1\;\mathrm{score}=\frac{2\times \mathrm{Precision}\times \mathrm{Recall}}{\mathrm{Precision}+\mathrm{Recall}}$$

In the aforementioned equations, TP represents the number of positive samples correctly identified by the model; TN represents the number of negative samples correctly identified; FP denotes the negative samples erroneously predicted as positive; and FN indicates the positive samples erroneously predicted as negative.

Beyond pixel-level accuracy, evaluating the computational efficiency of deep learning models is crucial for autonomous driving applications. To this end, this study additionally utilizes the number of parameters (Params) and the number of floating-point operations (FLOPs). The parameter count reflects the model’s spatial complexity, primarily comprising the learnable weights within the convolutional and fully connected layers. A smaller parameter count implies a reduced memory footprint and faster program initialization. Conversely, FLOPs measure the model’s computational time complexity by quantifying the total number of mathematical operations required for a single forward pass. By comprehensively considering these two metrics, we can effectively evaluate the trade-off between segmentation accuracy and computational efficiency, ensuring that the proposed EA-UNet is well-suited for real-time deployment on resource-constrained vehicle platforms. In practice, striking a reasonable balance between computational efficiency and recognition performance is essential to satisfy the demanding processing requirements of large-scale data [23].

### 4.3. Results Comparison and Analysis

To enhance the segmentation accuracy of the network and reduce computational training time, a transfer learning approach [24] was adopted prior to conducting the comparative experiments. Initially, the backbone network was pre-trained using the PASCAL VOC 2007 dataset. These pre-trained weights were then transferred to our model, facilitating a more rapid and stable convergence during training. Subsequently, the model was fine-tuned on our custom dataset to adapt perfectly to the specific binary classification task of left-turn lane detection.

Two distinct sets of experiments were designed to comprehensively evaluate the model’s performance. The first set comprises an ablation study, intended to isolate and verify the individual contributions of the proposed structural improvements (e.g., EfficientNet backbone and MP-ASPP module) on feature extraction accuracy. The second set involves comparative experiments, where the overall performance of the proposed EA-UNet is benchmarked against mainstream semantic segmentation architectures, specifically U-Net, DeepLabV3+, and PSPNet.

#### 4.3.1. Ablation Experiment

To systematically evaluate the impact of the proposed MP-ASPP module and the optimized backbone on model performance, a series of ablation experiments were conducted on the left-turn lane dataset. Using the standard U-Net as the baseline architecture, different experimental configurations were constructed by progressively integrating the improved backbone and the MP-ASPP module. Specifically, these configurations included: (a) the baseline U-Net; (b) the model with the encoder replacement; (c) the model with the MP-ASPP skip connection module; and (d) the proposed EA-UNet. The quantitative comparison results for these combinations are presented in Table 4.

Based on the ablation study results presented in Table 4, the following observations can be made:

(1) A comparison between Group A and Group B reveals that the original U-Net architecture suffers from excessive parameterization. By replacing the backbone, the parameter count and computational complexity (FLOPs) were reduced by 80.5% and 82.9%, respectively, albeit with a slight decrease in segmentation accuracy. This indicates that while backbone replacement involves a certain trade-off in performance, it achieves a substantial and necessary lightweighting effect.
(2) The data for Group C indicates that the integration of the MP-ASPP module enhances the mIoU, Precision, and F1-score by 4.5%, 3.08%, and 3.37%, respectively, compared to the baseline model. Notably, these performance gains were achieved with only a marginal increase in parameters and computational load. This demonstrates that the MP-ASPP module effectively boosts model performance with minimal computational overhead, highlighting its efficiency and cost-effectiveness in architectural optimization.
(3) In Group D, which incorporates both the optimized backbone and the MP-ASPP module, the mIoU, Precision, and F1-score reach 96.79%, 98.34%, and 98.35%, respectively, while maintaining a significantly reduced parameter count. These experimental results confirm that the synergistic integration of both improvements leads to a substantial enhancement in image segmentation performance. Consequently, the EA-UNet architecture successfully achieves high-precision semantic segmentation while simultaneously reducing model complexity, fulfilling the objective of a lightweight and efficient model.

#### 4.3.2. Comparative Experiment

To comprehensively validate the effectiveness of the proposed EA-UNet, its performance was benchmarked against three mainstream semantic segmentation networks: U-Net, DeepLabV3+, and PSPNet. To ensure a fair and rigorous comparison, all models were trained under identical hyperparameter configurations.

**Figure 8:**
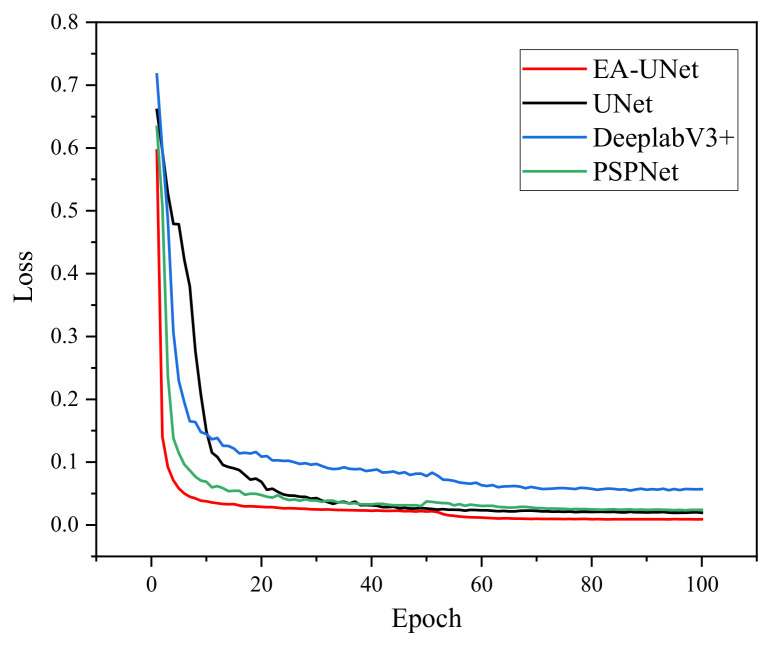
Comparison of network training loss.

The training loss trajectories for the different models are illustrated in Figure 8. A comparative analysis reveals that as the number of training epochs increases, the loss values for all four models gradually stabilize. Notably, the proposed EA-UNet exhibits a significantly faster and more stable convergence rate. The model effectively achieves convergence within approximately 20 epochs. This demonstrates superior training efficiency and substantially reduces the overall training cycle, further confirming the structural advantages of the optimized architecture.

The comprehensive quantitative comparison of the four networks is presented in Table 5. When applied to the left-turn lane segmentation task, the proposed EA-UNet demonstrates exceptional performance, achieving an mIoU of 96.79%, a Precision of 98.34%, and an F1-score of 98.35%.

Specifically, the proposed model outperforms U-Net, DeepLabV3+, and PSPNet by margins of 2.93%, 6.11%, and 4.71% in mIoU, respectively. Similar performance gains are observed in Precision (improvements of 2.33%, 4.45%, and 0.93%) and F1-score (improvements of 2.15%, 3.43%, and 2.60%). These enhancements are primarily attributed to the effective multi-scale feature extraction capabilities of the MP-ASPP module.

Furthermore, by integrating the lightweight EfficientNet-B0 backbone, EA-UNet achieves a significantly lower computational complexity compared to the baseline models. The parameter count is dramatically reduced to 9.121 M, and the FLOPs are compressed to 6.540 G. This represents a remarkable reduction of 79.23% in parameters and 81.44% in FLOPs relative to the original U-Net.

Finally, an evaluation of the inference speed (Frames Per Second, FPS) reveals that while U-Net reaches 64.22 FPS, the proposed EA-UNet maintains a highly competitive real-time inference speed of 60.23 FPS. Despite possessing merely one-fifth of the parameters and significantly lower computational complexity, our model achieves a far superior segmentation accuracy with only a negligible difference in processing speed. Ultimately, these comprehensive results rigorously demonstrate the superiority of the EA-UNet architecture, proving its capacity to achieve an optimal balance between lightweight efficiency and high-precision semantic segmentation.

#### 4.3.3. Segmentation Visualization Results

**Figure 9:**
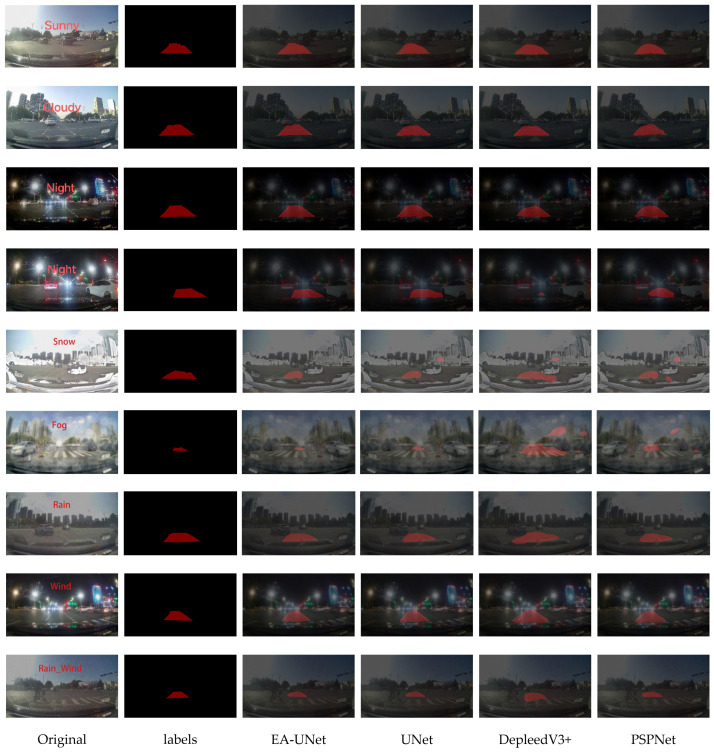
Comparison of network segmentation effects of different models.

To further validate the segmentation performance of the proposed network, a qualitative visual comparison was conducted against the baseline models. Four representative images were randomly selected from the test set, encompassing diverse weather conditions and varying degrees of occlusion over the left-turn lanes. The corresponding segmentation results are illustrated in Figure 9.

Visual comparisons reveal that while baseline models struggle with boundary smoothness and exhibit severe mis-segmentation under suboptimal illumination, the proposed EA-UNet maintains high consistency with ground-truth annotations. By effectively extracting fine details and preserving crisp edges, EA-UNet demonstrates superior robustness and generalization in complex scenarios.

**Figure 10:**
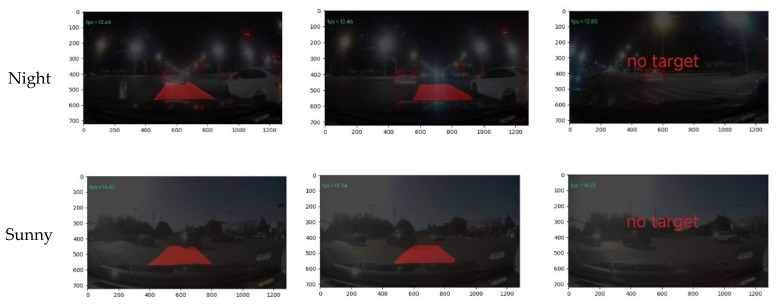
Video prediction segmentation effect.

To validate the real-world applicability of the proposed EA-UNet, video-based inference experiments were conducted across several complex environments. Two previously unseen video sequences—captured during daytime and nighttime—were selected for testing. The resulting segmentation outputs are illustrated in Figure 10. The results indicate that the algorithm accurately identifies both potential left-turn areas and non-target regions. High-precision segmentation is consistently maintained even under adverse conditions, such as degraded lane markings, uneven illumination, and vehicle occlusions. Notably, no instances of missed or false detections were observed throughout the sequences. Furthermore, the algorithm achieves an inference rate of approximately 10–15 FPS. While adapted for resource-constrained deployment, this processing speed is sufficient for stable real-time operation under low-speed driving conditions, ensuring reliable perception for autonomous navigation.

## 5. Conclusions

The proposed EA-UNet semantic segmentation algorithm achieves accurate identification of left-turn lanes and waiting areas, while demonstrating strong robustness under complex environmental conditions, including shadow occlusion, lane degradation, and variable weather. These results indicate that EA-UNet constitutes an effective and lightweight semantic segmentation framework for structured road-scene understanding. By strategically integrating an EfficientNet-B0 encoder, the proposed MP-ASPP module, and the CBAM attention mechanism, the network effectively addresses several inherent limitations of the conventional U-Net architecture, including high computational cost, insufficient segmentation accuracy, and coarse boundary delineation.

Compared with mainstream semantic segmentation methods, the proposed model delivers substantial improvements in both segmentation performance and computational efficiency. Specifically, EA-UNet achieves an mIoU of 96.79%, a Precision of 98.34%, and an F1-score of 98.35%, corresponding to improvements of 2.93, 2.33, and 2.15 percentage points over the baseline U-Net, respectively. In addition, the number of parameters and FLOPs are reduced by 79.23% and 81.44%, respectively, highlighting the lightweight nature of the proposed architecture. Despite this significant reduction in model complexity, the algorithm maintains an inference speed of 60.23 FPS, thereby satisfying the real-time perception requirements of autonomous driving systems.

Real-vehicle field experiments further verify the practical applicability of the proposed approach in low-speed left-turn waiting scenarios. Even when trained on a relatively limited dataset of static images, the model is able to identify operational regions accurately and efficiently in complex road environments, demonstrating promising real-world deployment potential. Moreover, the proposed architecture may be extended beyond left-turn scenarios to a wider range of road perception tasks involving severe lane-marking occlusion, pavement damage, or challenging illumination conditions. Overall, the results confirm that EA-UNet provides a favorable balance between segmentation accuracy, computational efficiency, and deployment feasibility for intelligent driving applications.

## Figures and Tables

**Table 1:** Improved EfficientNet-B0 network parameter structure.

Stage	Parameter	Number of Channels	Number of Layers	Resolution
1	Conv [3 × 3] & BN & Swish	32	1	224 × 224
2	MBconv1, 3 × 3	16	1	112 × 112
3	MBconv6, 3 × 3	24	2	112 × 112
4	MBconv6, 5 × 5	40	2	56 × 56
5	MBconv6, 3 × 3	80	3	28 × 28
6	MBconv6, 5 × 5	112	1	14 × 14
7	MBconv6, 5 × 5	192	2	14 × 14
8	MBconv6, 3 × 3	320	1	7 × 7

**Table 2:** Configuration of experimental hardware environment.

Related Configurations	Configure Parameters
Operation system	Windows 10 Pro
Processor	Intel(R) Core(TM) i7-9700 CPU @ 3.00 GHz 3.00 GHz
Internal memory	32.0 GB
Graphics card	NVIDIA GeForce GTX 1660 Ti
Video memory	6G

**Table 3:** Configuration of experimental software environment.

Related Configurations	Configure Parameters
Programming language	Python3.9.10
Deep Learning Framework	Pytorch
GPU computing platform	CUDA 12.2
Optimizer	Adam
Learning rate	0.0001

**Table 4:** Ablation results.

Ablation Group	Improved Encoder	MP-ASPP	MIOU	Precision	F1 Score	Parameters/MB	FLOPs/G
a	×	×	93.86	96.01	96.2	43.933	35.238
b	√	×	92.12	96.08	95.42	8.561	6.017
c	×	√	98.36	99.09	99.57	44.493	35.761
d	√	√	96.79	98.34	98.35	9.121	6.540

**Table 5:** The overall classification performance of each model on the test set.

Models	MIOU	Precision	F1 Score	Params (M)	FLOPs (G)	FPS
**DepleedV3+**	90.68	93.89	94.92	54.709	31.935	44.95
**PSPNet**	92.08	97.41	95.75	46.716	118.447	28.76
**UNet**	93.86	96.01	96.2	43.933	35.238	64.22
**EA-UNet**	96.79	98.34	98.35	9.121	6.540	60.23

## Data Availability

The data presented in this study are available on request from the corresponding author.
